# Knowledge, Attitudes and Practices of Audiologists in India Towards Audiological Care of Individuals with Cognitive Impairments—A Preliminary Exploratory Study

**DOI:** 10.3390/audiolres16050121

**Published:** 2026-08-23

**Authors:** Dhanshree R. Gunjawate, Poorva Advait Patki, Twinkle Lijo Kirianthan, Rohit Ravi

**Affiliations:** Department of Audiology and Speech Language Pathology, Kasturba Medical College Mangalore, Manipal Academy of Higher Education, Manipal 576104, India; dhanshree.g@manipal.edu (D.R.G.);

**Keywords:** cognitive impairments, hearing loss, India, audiologists

## Abstract

**Background/Objectives**: The present study aimed to explore the knowledge, attitudes and practices of audiologists in India towards audiological care of individuals with cognitive impairments. **Methods**: The questionnaire was adapted from a previously published questionnaire designed to explore knowledge, attitude, and practices regarding comorbid hearing loss and cognitive impairment among Australian allied hearing healthcare professionals. Participants included audiologists with at least a bachelor’s degree in audiology and speech-language pathology, a minimum of one year of work experience and registration with the Rehabilitation Council of India. An online questionnaire was created using Google Forms and disseminated via social media and personal emails. Continuous variables were summarized using means and standard deviation, while categorical variables were reported as frequencies and percentages. **Results**: A total of 140 audiologists participated in the study. Overall, the responses indicated a generally positive awareness towards managing individuals with comorbid hearing loss and cognitive impairment. Most respondents were mindful about the coexistence of hearing loss and cognitive impairment while working with older adults, with 74.3% either agreeing or strongly agreeing. This self-reported awareness was not consistently reflected in reported practice, with only 31.4% having used a formal cognitive screening test as part of their clinical practice. **Conclusions**: Overall, the findings suggest that while audiologists report a degree of self-perceived awareness regarding cognitive impairment, this does not consistently translate into practice. Notable gaps remain in training, awareness of appropriate screening tools, and actual implementation of cognitive screening, highlighting the need for greater clinical support, structured training, and clearer referral pathways to bridge the gap between self-reported awareness and evidence-based practice.

## 1. Introduction

Cognition refers to a range of mental processes associated with knowing and awareness, including comprehension, conception, decision-making, judgement, imagination, language, problem-solving, perception, planning, remembering, reasoning, and visuospatial abilities [1,2]. A cognitive impairment or deficit refers to a dysfunction in one or more cognitive domains and can range from mild to severe. As per the *Diagnostic and Statistical Manual of Mental Disorders 5th edition* (DSM-5), conditions such as dementia, delirium, amnesia, and other cognitive disorders have been categorized under neurocognitive disorders, which now include delirium, major neurocognitive disorders, and mild neurocognitive disorders. Delirium refers to an acute and fluctuating alteration in awareness, attention, and cognition. Major neurocognitive disorders (dementia) refer to an overall decline in memory function and other cognitive skills that impacts a person’s ability to perform day-to-day activities. Minor neurocognitive disorders, also termed mild cognitive impairment (MCI), involve a modest cognitive decline in one or more cognitive domains that may not interfere with independence in activities of daily living; however, they require increased time, effort, accommodation, and compensatory strategies [3,4].

The global prevalence of mild cognitive impairment varies between 14% and 18% among individuals aged 70 years or older [5,6] and from 3% to 42% among adults aged 60 years and above [7], with an overall prevalence estimate of 15.56% [8]. The wide and overlapping ranges should not be regarded as directly comparable global figures, as they originate from heterogeneous studies with varying population characteristics, diagnostic criteria, and assessment methods. Notably, the absence of standardized definitions and the evolving terminology for mild cognitive impairment across studies contribute to significant variability in reported prevalence, thereby complicating precise estimation of the associated social burden [7].

The World Health Organization, in the position paper ‘Optimizing Brain Health Across the Life Course,’ has provided a conceptual framework to optimize brain health throughout the life course through physical health, safety and security, healthy environments, learning, social connections, and access to quality services [9]. Further, the World Health Organization has also provided guidelines named ‘risk reduction of cognitive decline and dementia’ [10]. The actions recommended in these guidelines can help delay or prevent cognitive impairment. These include modifying lifestyles, encouraging healthcare systems and governments to adopt prevention-oriented strategies, and helping to support health professionals and policymakers in best practices for risk reduction.

Over 5% of the world’s population requires rehabilitation to address their hearing loss, with a projection that by 2050, one in every 10 individuals will be affected. Further, nearly 80% of these individuals live in low- and middle-income countries. As age increases, the prevalence of hearing loss also increases; among individuals aged 60 and older, more than 25% experience disabling hearing loss [11]. Along with limitations in communication, social isolation, stigma, impact on economy, access to education and employment, untreated hearing loss can adversely affect cognition [12], with older adults experiencing increased odds of depression [13]. The higher prevalence of hearing loss is skewed towards middle- and low-sociodemographic-index regions and is linked to population growth and ageing [14,15]. An association between hearing loss, cognitive impairment [16,17,18], and dementia [19,20,21] has also been reported. Timely intervention through hearing aids and cochlear implants helps reduce cognitive deterioration [22,23,24].

Recent studies have encouraged the inclusion of cognitive screening in hearing assessments and hearing screening in cognitive assessments [25,26,27]. The inclusion of cognitive screening in hearing assessments helps to identify cognitive deficits that may influence hearing assessment results and vice versa. Globally, professional organizations [28,29] are increasingly emphasizing the importance of incorporating cognitive screening tests in audiological testing and practice while providing comprehensive patient care. Studies in Australia [30] and China [31] have also identified gaps in the knowledge, attitudes, and practices among hearing healthcare professionals in addressing the complexities of comorbid hearing loss and cognitive assessment. Certain related aspects needed additional focused training to further empower and upskill these professionals. This would enable better services to be provided to elderly individuals with comorbid cognitive issues and hearing loss. A similar exploration has not yet been conducted in the Indian context, and several context-specific factors justify this investigation.

The field and curriculum of audiology and speech-language pathology in India are regulated by the Rehabilitation Council of India (RCI). Nevertheless, variations persist among institutions and clinical settings regarding clinical practices, training exposure, resources, and access to interdisciplinary healthcare services. Additionally, access to specialist referral pathways is often limited in rural and resource-constrained settings. These disparities may affect how audiologists identify, manage, and refer clients with suspected cognitive impairment, compared with healthcare systems with more established interdisciplinary referral networks. Furthermore, cultural perceptions of ageing, family-based caregiving practices, and healthcare-seeking behaviours may further impact client presentation and clinical decision-making in India. Consequently, findings from studies conducted in Australia, China, or other countries may not be directly applicable to Indian audiological practice, highlighting the necessity for context-specific evidence from India.

The diversity of healthcare services and differences in curriculum and awareness levels highlight the need for an investigation in this context. Examining the knowledge, attitudes, and practices of audiologists in India towards audiological care of individuals with cognitive impairments will help identify current gaps and help plan specific targeted training initiatives which will further help in developing guidelines for clinical practices in the Indian context. The present study aimed to explore the knowledge, attitudes and practices of audiologists in India towards audiological care of individuals with cognitive impairments. The effects of cognitive deficits among patients and their impact on clinical practices, awareness of different cognitive screening tools, referral pathways, and the inclusion of family members were included in the knowledge section. The section on attitudes included items examining perceptions of their role in identifying cognitive deficits, confidence in managing clinical populations, and any challenges faced. The practices section included items related to conducting cognitive screening tests, preferred test types, and discussing specific needs and requirements of patients with suspected cognitive deficits. The last section included items related to training and training preferences.

## 2. Materials and Methods

A cross-sectional online survey was conducted to explore the knowledge, attitudes, and practices of audiologists in India towards audiological care of individuals with cognitive impairments. The study was conducted in accordance with the principles of the Declaration of Helsinki and received ethical approval from the Institutional Ethics Committee of Kasturba Medical College, Mangalore (IECKMCMLR09/2025/568). Only the participants who consented to the electronic consent form could participate in the study.

### 2.1. Knowledge, Attitudes and Practices Questionnaire

The questionnaire was adapted from a previously published questionnaire exploring knowledge, attitudes, and practices regarding comorbid hearing loss and cognitive impairment developed for Australian allied hearing healthcare professionals [30]. The original questionnaire was used with minimal modifications. ‘Allied hearing healthcare professionals’ was replaced by ‘audiologists’ to specify the target population. A question about community support services in Australia was removed because it was not contextually appropriate. An open-ended question on decision-making for the cognitive screening test was excluded to reduce the response burden and maintain closed-ended format for all questions. Finally, a question on engagement with general practitioners was removed due to differences in the clinical referral pathways. Any reworded questions were reviewed by the three audiologists, each with a minimum of ten years of clinical and research experience, to ensure the relevance, clarity, and conceptual accuracy of the content. This expert review process was undertaken to establish the content validity of the questionnaire prior to distribution. Additionally, content validity was assessed using the Scale-Content Validity Index (S-CVI) [32].

The final questionnaire was administered in English and comprised the following domains: demographic details, knowledge, attitude, and practices towards audiological care of individuals with cognitive impairments and training.

### 2.2. Participants

Participants included professional audiologists practising in India. Audiologists with a minimum of a bachelor’s degree in audiology and speech-language pathology, with a minimum of one year of work experience and registered under the Rehabilitation Council of India were included. Those studying and working outside India were excluded. At a 95% confidence level (α = 0.05, z = 1.96), with a margin of error of 6.5% and an estimated population proportion of 81% (*p* = 0.81, q = 0.19), the sample size was determined using the single population proportion formula, resulting in an estimated sample size of 140.

### 2.3. Data Collection

The final online questionnaire was created using Google Forms and distributed through social media posts and personal emails. The contact details of the audiologist were obtained from the Rehabilitation Council of India website, and email requests for participation were sent. Additionally, survey flyers with study details and a Google Forms link were circulated via social media pages.

The Google Forms link included information about the study, informed consent, and the questionnaire. Only participants who consented could access the questionnaire. No personal identifiers such as full name, phone number, or email address were collected to maintain anonymity. Audiologists who consented could access the questionnaire and had to click on submit to submit their responses. Audiologists who did not consent could not participate and were redirected to the final section, where they could exit the questionnaire without completing it. The responses were saved directly on Google Drive and could be accessed only by the investigators. The respondents took approximately 10–12 min to complete the questionnaire. Google Forms settings are configured to allow only a single response per individual. This measure is implemented to reduce the likelihood of duplicate submissions. Further, during the data cleaning process, responses were screened for duplicate timestamps and highly similar response patterns, and no such duplicate responses were identified.

### 2.4. Analysis

Content validity was assessed using the Scale-Content Validity Index (S-CVI) [32]. A score of 0.80 was obtained for the S-CVI, indicating excellent content validity of the questionnaire. Continuous variables were summarized using means and standard deviation, while categorical variables were reported as frequencies and percentages. Spearman’s rank correlation was used to examine the relationships between age, professional experience, and confidence-related items. Pearson chi-square test was used to examine the association between prior training and use of formal cognitive screening tests, with Cramér’s V calculated as a measure of effect size. All statistical analyses were done using IBM SPSS Statistics Version 25 [33].

## 3. Results

### 3.1. Demographic Details of Participants

One hundred and forty audiologists participated in the study. As the survey was distributed via social media, the total number of individuals who were reached or had access to the survey could not be determined. Therefore, a response rate could not be calculated. The survey platform Google Forms mandated the completion of all items prior to submission, thereby ensuring no missing data.

The demographic details have been depicted in Table 1.

### 3.2. Knowledge Towards Audiological Care of Individuals with Cognitive Impairment

Eleven items explored the knowledge towards audiological care of individuals with cognitive impairment, to be rated on a five-point scale (strongly agree, agree, neutral, disagree, strongly disagree). The responses are shown in Table 2.

Overall, the responses indicated a generally positive awareness towards managing individuals with comorbid hearing loss and cognitive impairment. A large majority (92.1%) indicated that while assessing an individual with cognitive impairment, they spend more time and provide extra support. Additionally, 85% agreed or strongly agreed that supplemented materials, such as written and/or visual materials, are needed. Family members provided valuable information during hearing assessments regarding hearing loss and cognitive functioning, as per 89.3% respondents. Three-quarters of the respondents (75%) either strongly agreed or agreed that they were able to identify whether a client has cognitive impairment.

Most respondents were mindful about the coexistence of hearing loss and cognitive impairment while working with older adults, with 74.3% either agreeing or strongly agreeing. Similarly, 73.5% agreed or strongly agreed that objective hearing assessments can be used for individuals with comorbid hearing loss and cognitive impairment. Awareness of referral pathways was moderate to high. Two-thirds of respondents (66.4%) reported being aware of ways to initiate referrals for further cognitive assessment while a slightly higher proportion (75%) reported being aware of referral pathways for hearing-related investigations.

The responses related to specific knowledge of cognitive screening and tools were scattered with relatively few respondents choosing to agree or strongly agree. Only 55.8% agreed or strongly agreed that there are specifically designed cognitive screening tests for people with hearing loss, while 37.1% selected neutral. Only 40% reported having the training and expertise to administer and interpret the results of a brief cognitive screening test. A total of 57.1% respondents reported having the knowledge needed to formally support patients’ cognitive needs while making hearing rehabilitation plans.

### 3.3. Attitudes Towards Audiological Care of Individuals with Cognitive Impairment

Seven items explored the attitude towards audiological care of individuals with cognitive impairment. The responses of five items that were rated on a five-point scale (strongly agree, agree, neutral, disagree, strongly disagree) have been depicted in Table 3.

Overall, the respondents demonstrated positive attitudes towards incorporating cognitive considerations during audiological care. A large majority (85.7%) strongly agreed or agreed that there is value in asking an older client about memory issues during the assessment. Further, 75% reported being confident and asking older clients if they have memory issues, while 67.1% reported being confident in having in-depth discussions with an older client about their memory issues. Age and professional experience were not significantly correlated with confidence in asking older clients about memory issues (*p* = 0.151 and *p* = 0.328, respectively) or confidence in having in-depth discussions about memory issues (*p* = 0.544 and *p* = 0.816, respectively).

**Figure 1:**
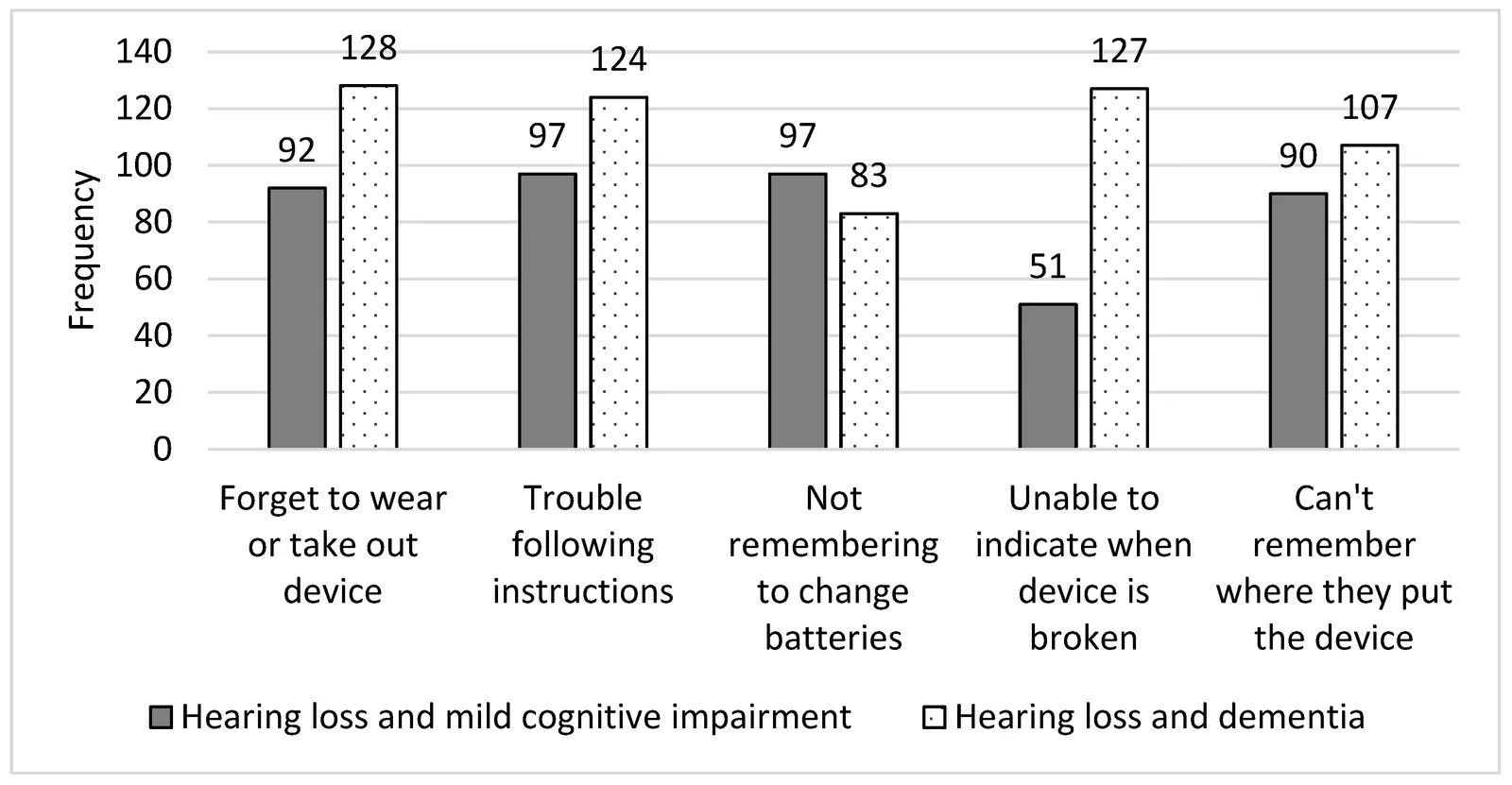
Challenges faced by individuals with mild cognitive impairment and individuals with mild cognitive impairment with hearing loss.

More than 80% of the respondents strongly agreed or agreed that managing clients with cognitive impairments can be challenging. About 75% participants also strongly advocated the professional role of audiologists in identifying cognitive impairment in older adults with hearing loss, while 23.6% were neutral. For the next two items under attitudes, the respondents were provided with a list of statements about the challenges faced by individuals with mild cognitive impairment and hearing loss with dementia. Their responses have been depicted in Figure 1.

### 3.4. Practices in Audiological Care of Individuals with Cognitive Impairment

Thirteen items explored the practices in audiological care of individuals with cognitive impairment. The responses to the seven items that were rated on a five-point scale (never, rarely, sometimes, often, always) have been depicted in Table 4.

The audiologists’ responses ranged from moderate (sometimes) to frequent (always) engagement in the included clinical practices. About 50% of the respondents indicated that they often or always discuss the link between hearing loss and cognitive impairment with older clients, while 31.4% do it sometimes. Similarly, 53.5% reported often or always asking clients and/or their carers about cognitive functioning while 37.1% did so sometimes.

Regarding access to tools and strategies to support hearing-impaired clients with cognitive impairment, respondents were divided in their views. Approximately equal proportions disagreed (20.7%) and agreed (25%) that they had the necessary tools, while the largest proportion remained neutral (41.4%), suggesting uncertainty or inconsistency in access to appropriate resources and training. In contrast, perceptions of workplace support for allocating additional appointment time were predominantly negative. More than half of the respondents (55.7%) disagreed that their workplace provided such support, while only 11.4% agreed.

The choice of deciding to do a cognitive screening test on older clients was based on client reporting memory issues (69.9%), carer/family member reporting memory issues (66.4%), inconsistent hearing assessment results (52.1%) and client’s age (37.1%). Although 47.2% of respondents indicated on the Likert-scale item that they ‘sometimes,’ ‘often,’ or ‘always’ conduct formal cognitive screening tests on older hearing-impaired clients as part of their routine practice (Table 4), only 31.4% (n = 44) reported having ever used a specific, named formal cognitive screening tool. Mini-Mental State Examination was most popular (n = 32) followed by Montreal Cognitive Assessment (n = 29). The less popular options were Montreal Cognitive Assessment for the Hearing-Impaired (n = 9) and General Practitioner Assessment of Cognition (n = 4). This discrepancy may reflect variability in respondents’ interpretation of formal screening, with some considering informal or non-standardized cognitive checks equivalent to formal screening, even if they do not use a validated named instrument. A significant association was noted between having received training for supporting clients with memory issues or cognitive impairments and use of cognitive screening tests (χ^2^ = 17.1, df = 1, *p* < 0.001, effect size 0.35). For the statement, ‘If I suspect that a hearing-impaired client has cognitive impairment as well, I will refer to specialists’, 90.7% replied in agreement.

When asked to indicate preferred forms of training that would empower them to work with clients with hearing loss and cognitive impairment, respondents most frequently selected clinical guideline/tipsheets (n = 69, 49.3%) followed by in-person course/workshop (n = 64, 45.7%) and live online course/workshop (n = 64, 45.7%). The least preferred was books and journal articles (n = 31, 22.1%). The most preferred content for empowerment to work with clients with hearing loss and cognitive impairment was strategies for assessment and rehabilitation (n = 127, 90.7%). The responses for other contents were as follows: talking about memory loss with hearing-impaired clients (n = 64, 45.7%), distinguishing between memory and behavioural issues (n = 52, 37.1%), and background information about hearing loss and dementia (n = 39, 27.9%).

## 4. Discussion

The present study explored the knowledge, attitudes, and practices of audiologists in India towards audiological care of individuals with cognitive impairments. Overall, the audiologists demonstrated good awareness of the relationship between hearing loss and cognitive impairment and expressed positive attitudes toward their role in caring for this population. However, important gaps were identified in knowledge related to cognitive screening, referral pathways, and the routine implementation of cognitive assessment, indicating the need for additional professional training.

Most respondents recognized that hearing loss frequently coexists with cognitive impairment and agreed that individuals with cognitive impairment require additional consultation time, alternative assessment approaches, and enhanced communication strategies. These findings are consistent with those reported by [30] who observed that Australian hearing healthcare professionals demonstrated good awareness of the association between hearing loss and cognitive impairment and routinely adapted clinical management for cognitively vulnerable patients. In contrast to the Australian context, where standardized referral pathways to geriatricians and neurologists are well-established within a unified public healthcare system, Indian audiologists function within a highly fragmented healthcare landscape. This landscape includes private clinics, corporate hospitals, government institutions, and academic centres, each offering varying levels of access to interdisciplinary referral networks. This structural heterogeneity may account for the discrepancy between the positive attitudes toward interdisciplinary collaboration observed in both the Australian and current studies and the actual practice patterns across different settings. The increasing awareness among audiologists is likely attributable to the growing evidence demonstrating hearing loss as one of the most important modifiable risk factors for dementia [34,35]. Longitudinal studies [19,21,36] further demonstrated that untreated hearing loss is associated with a significantly increased risk of cognitive decline and incident dementia, reinforcing the importance of early identification and intervention.

Although general awareness was high, participants demonstrated limited confidence in cognitive screening and cognitive rehabilitation planning. Less than half reported having adequate training to administer and interpret cognitive screening tests, while many were uncertain about hearing-adapted cognitive assessment tools. Ref. [37] identified a similar need reported by healthcare professionals for more training to detect and manage coexistence of cognitive and sensory impairments, and a need for correctly validated assessment tools for vision, hearing and cognition testing. Ref. [30] reported similar deficiencies in knowledge related to cognitive screening tools and referral pathways despite overall positive knowledge regarding cognitive impairment. These findings collectively suggest that while audiologists appreciate the clinical importance of cognition, formal education has not adequately equipped them to incorporate cognitive screening into routine practice. The training deficit is exacerbated by the lack of a standardized audiology clinical curriculum throughout India. Entry-level qualifications vary from diploma-level to master’s-level training across institutions with differing accreditation standards. In contrast to countries with centralized professional regulatory bodies that mandate continuing education on emerging clinical competencies, such as cognitive screening, the Indian audiology workforce lacks equivalent formal specializations or sub-specialty certifications in geriatric or cognitive-hearing audiology.

Participants also demonstrated positive attitudes toward discussing memory concerns with older adults and acknowledged that audiologists have an important role in identifying cognitive impairment. These findings are encouraging because positive professional attitudes are essential for implementing expanded models of patient-centred care. Similar attitudes have been reported in previous studies, where audiologists supported interdisciplinary collaboration between audiology, geriatrics, neurology, and primary care to improve the management of older adults with hearing loss and cognitive impairment [30,37]. However, despite these positive attitudes, relatively few respondents routinely performed formal cognitive screening, indicating a gap between knowledge and clinical practice.

In clinical practice, audiologists frequently reported providing written or visual instructions, involving family members during appointments, and allocating additional consultation time for patients with suspected cognitive impairment. These findings align with recommendations from [26], who emphasized adapting communication strategies to compensate for cognitive limitations and improve hearing rehabilitation outcomes. Similarly, ref. [27] recommended that audiologists modify testing procedures and consider cognitive screening when behavioural hearing assessment results appear inconsistent. Family-centred care has also been recognized as an important component of hearing rehabilitation because caregivers often provide valuable information regarding patients’ functional communication, memory, and adherence to hearing aid use.

Despite these positive adaptations, the use of formal cognitive screening remained limited. Most participants relied on subjective reports from patients or caregivers rather than standardized cognitive screening instruments. Comparable findings were reported by [30], where informal questioning was considerably more common than objective cognitive assessment. This practice may reduce opportunities for identifying mild cognitive impairment during its earliest stages. Several authors have therefore advocated incorporating hearing-appropriate cognitive screening tools, such as the Hearing-Impaired Montreal Cognitive Assessment (HI-MoCA), into audiological practice to improve early detection while minimizing the confounding effects of hearing loss on cognitive test performance [27,38].

Participants also identified a need for additional education, particularly practical clinical guidelines, workshops, and assessment strategies for patients with hearing loss and cognitive impairment. Similar educational needs have been consistently reported across studies [30,37]. As hearing loss has become an established modifiable risk factor for dementia [35], audiologists are increasingly expected to contribute to early detection, counselling, and multidisciplinary referral. Developing structured continuing professional education, incorporating cognitive health into audiology curricula, and establishing evidence-based national clinical guidelines may therefore improve clinicians’ confidence and facilitate more consistent integration of cognitive assessment into routine audiological care. These findings collectively indicate the necessity for coordinated action across various levels. Specifically, curriculum reform should be implemented at the institutional level; scope-of-practice clarification is required at the regulatory level, and resource allocation and tool development must occur at the health-system level. This approach is preferable to relying solely on the motivation of individual practitioners to bridge the awareness–practice gap. Further, the association between hearing loss and cognitive impairment is likely multifactorial, extending beyond cognitive load, reduced communication, and social isolation. Emerging evidence suggests that impaired glymphatic clearance may represent a shared pathophysiological pathway linking auditory dysfunction, ageing, neuroinflammation, and cognitive decline [39]. These also need to be incorporated in the curriculum and clinical practice.

This study has several limitations that should be acknowledged. Participants were recruited through convenience sampling, which limits the generalizability of the findings to a broader population and may have introduced some selection bias. The data were collected using self-reported measures, which can lead to social desirability and recall bias. The study findings may be subject to non-response bias, as opinions of individuals who chose not to participate may differ from those who did. There was no assessment of actual clinical practice to ascertain how much of what is reported is actually being implemented.

Also, the sample was predominantly composed of young participants, which restricts the applicability of the findings to older age groups and limits generalizability across the broader population. The cross-sectional study design limits any conclusions regarding causality. Future research should address these limitations by employing incorporating objective measures where feasible, including a more age-diverse sample, and utilizing longitudinal designs.

## 5. Conclusions

Overall, the findings suggest that audiologists have positive knowledge and attitudes regarding cognitive impairment but require greater clinical support to translate this awareness into routine evidence-based practice. Strengthening interdisciplinary collaboration, improving access to hearing-specific cognitive screening tools, and expanding continuing professional education may ultimately improve the quality of care provided to older adults with hearing loss and cognitive impairment.

## Figures and Tables

**Table 1:** Demographic details.

		Mean ± SD	Range
Age (in Years)		27.7 ± 4.82	21 to 47 years
Experience (in Years)		4.38 ± 4.47	1 to 25 years
		**n**	**%**
Gender	Male	71	50.7
	Female	69	49.3
Degree	Bachelors	52	37.1
	Masters	78	55.7
	PhD	10	7.1
Roles at workplace	Hearing assessment	139	99.3
	Vestibular assessment	59	42.1
	Hearing aid fitting	130	92.9
	Cochlear implant fitting	24	17.1
	Auditory verbal therapy	43	30.7
	Intra-operative monitoring	18	12.9
	Counselling	120	85.7
	Research	53	37.9
	Teaching	46	32.9
	Others	12	8.6

**Table 2:** Knowledge towards audiological care of individuals with cognitive impairment.

	Strongly Agree	Agree	Neutral	Disagree	Strongly Disagree
	n (%)				
When working with older adults, I am mindful that they may have hearing loss and cognitive impairment	39 (27.9%)	65 (46.4%)	26 (18.6%)	7 (5%)	3 (2.1%)
There are objective hearing assessments that can be used for people who may have cognitive impairment	31 (22.1%)	72 (51.4%)	28 (20%)	8 (5.7%)	1 (0.7%)
Assessing someone with a cognitive impairment means I might need to spend more time and provide alternative testing and extra support to the client	58 (41.4%)	71 (50.7%)	7 (5%)	1 (0.7%)	3 (2.1%)
There are specifically designed cognitive screening tests that can be used for people with hearing loss	18 (12.9%)	60 (42.9%)	52 (37.1%)	9 (6.4%)	1 (0.7%)
I have the training and expertise to administer and interpret the results of a brief cognitive screening test	8 (5.7%)	48 (34.3%)	56 (40.0%)	24 (17.1%)	4 (2.9%)
Instructions for how to use hearing devices for people with cognitive impairment should be supplemented by written and/or visual form	53 (37.9%)	66 (47.1%)	15 (10.7%)	4 (2.9%)	2 (1.4%)
I know how to formally support the cognitive needs of patients when making hearing rehabilitation plans (for example, which cognitive tests to use, how to use objective hearing tests, etc.)	14 (10%)	66 (47.1%)	44 (31.4%)	14 (10%)	2 (1.4%)
I am aware of how to initiate appropriate formal referral pathways for clients who have both hearing loss and cognitive impairment and need further investigation of their memory	27 (19.3%)	66 (47.1%)	33 (23.6%)	10 (7.1%)	4 (2.9%)
I am aware of how to initiate appropriate formal referral pathways for clients who have both hearing loss and cognitive impairment and need further investigation of their hearing loss	29 (20.7%)	76 (54.3%)	23 (16.4%)	10 (7.1%)	2 (1.4%)
Family members can provide valuable information regarding the person with hearing loss and their cognitive functioning, and there is value in them attending a hearing appointment	65 (46.4%)	60 (42.9%)	8 (5.7%)	4 (2.9%)	3 (2.1%)
I am able to identify whether a client has cognitive impairment	30 (21.4%)	75 (53.6%)	30 (21.4%)	4 (2.9%)	1 (0.7%)

**Table 3:** Attitudes towards audiological care of individuals with cognitive impairment.

	Strongly Agree	Agree	Neutral	Disagree	Strongly Disagree
	n (%)				
I see value in asking an older client about memory issues as part of my assessment.	40 (28.6%)	80 (57.1%)	17 (12.1%)	1 (0.7%)	2 (1.4%)
I feel confident asking an older client if they have memory issues.	33 (23.6%)	72 (51.4%)	28 (20%)	5 (3.6%)	2 (1.4%)
I feel confident having an in-depth discussion with an older client about their memory issues.	28 (20%)	66 (47.1%)	37 (26.4%)	6 (4.3%)	3 (2.1%)
Managing clients with cognitive impairment can be challenging.	40 (28.6%)	76 (54.3%)	19 (13.6%)	4 (2.9%)	1 (0.7%)
Audiologists have a role in identifying cognitive impairment in older adults with hearing loss.	37 (26.4%)	69 (49.3%)	33 (23.6%)	0	1 (0.7%)

**Table 4:** Practices in audiological care of individuals with cognitive impairment.

	Never	Rarely	Sometimes	Often	Always
I talk to older clients about the link between hearing loss and cognitive impairment.	4 (2.9%)	18 (12.9%)	44 (31.4%)	44 (31.4%)	30 (21.4%)
I specifically ask older clients and/or their carers or family members questions about the client’s cognitive functioning.	1 (0.7%)	12 (8.6%)	52 (37.1%)	44 (31.4%)	31 (22.1%)
I recommend objective hearing assessments if I suspect that a client’s cognitive impairment is affecting their hearing test results.	8 (5.7%)	22 (15.7%)	39 (27.9%)	38 (27.1%)	33 (23.6%)
I conduct formal cognitive screening tests with older, hearing-impaired clients as part of my practice.	29 (20.7%)	45 (32.1%)	40 (28.6%)	18 (12.9%)	8 (5.7%)
I talk to clients about how their cognitive impairment could impact their hearing rehabilitation.	4 (2.9%)	14 (10%)	45 (32.1%)	45 (32.1%)	32 (22.9%)
For hearing-impaired clients with cognitive impairment, I provide instructions/information about how to use their hearing devices in writing or in video format.	4 (2.9%)	13 (9.3%)	48 (34.3%)	38 (27.1%)	37 (26.4%)
I allocate extra time in my consultation to support hearing-impaired clients with suspected cognitive impairments.	2 (1.4%)	10 (7.1%)	36 (25.7%)	48 (34.3%)	44 (31.4%)

## Data Availability

The data related to the results of this study can be obtained from the corresponding authors upon reasonable request.

## Abbreviations

The following abbreviations are used in this manuscript:

S-CVI	Scale-Content Validity Index
HI-MoCA	Hearing-Impaired Montreal Cognitive Assessment
